# Pedunculopontine Nucleus Degeneration Contributes to Both Motor and Non-Motor Symptoms of Parkinson’s Disease

**DOI:** 10.3389/fphar.2019.01494

**Published:** 2020-01-15

**Authors:** Nicole Elaine Chambers, Kathryn Lanza, Christopher Bishop

**Affiliations:** Department of Psychology, Binghamton University, Binghamton, NY, United States

**Keywords:** Parkinson’s disease, pedunculopontine nucleus, neurodegeneration, gait deficits, non-motor symptoms, REM sleep behavior disorder, pharmacology

## Abstract

Parkinson’s disease (PD) is a neurodegenerative disorder characterized by hypokinetic motor features; however, patients also display non-motor symptoms like sleep disorders. The standard treatment for PD is dopamine replacement with L-DOPA; however, symptoms including gait deficits and sleep disorders are unresponsive to L-DOPA. Notably, these symptoms have been linked to aberrant activity in the pedunculopontine nucleus (PPN). Of late, clinical trials involving PPN deep brain stimulation (DBS) have been employed to alleviate gait deficits. Although preclinical evidence implicating PPN cholinergic neurons in gait dysfunction was initially promising, DBS trials fell short of expected outcomes. One reason for the failure of DBS may be that the PPN is a heterogenous nucleus that consists of GABAergic, cholinergic, and glutamatergic neurons that project to a diverse array of brain structures. Second, DBS trials may have been unsuccessful because PPN neurons are susceptible to mitochondrial dysfunction, Lewy body pathology, and degeneration in PD. Therefore, pharmaceutical or gene-therapy strategies targeting specific PPN neuronal populations or projections could better alleviate intractable PD symptoms. Unfortunately, how PPN neuronal populations and their respective projections influence PD motor and non-motor symptoms remains enigmatic. Herein, we discuss normal cellular and neuroanatomical features of the PPN, the differential susceptibility of PPN neurons to PD-related insults, and we give an overview of literature suggesting a role for PPN neurons in motor and sleep deficits in PD. Finally, we identify future approaches directed towards the PPN for the treatment of PD motor and sleep symptoms.

## Introduction

### Cellular, Molecular, and Neuroanatomical Properties of Pedunculopontine Tegmental Nucleus

**Figure 1:**
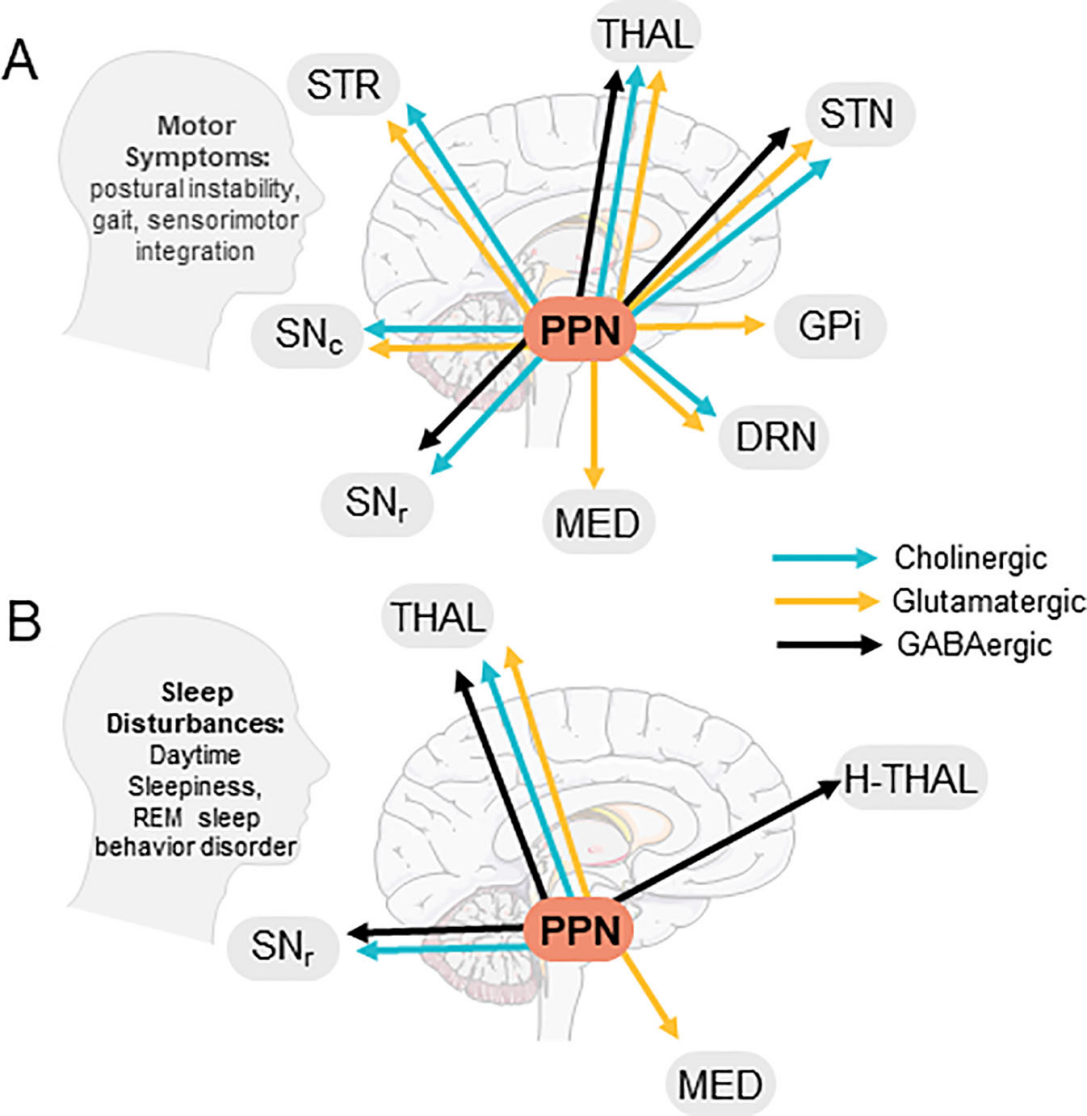
Efferent PPN projections and putative involvement in PD symptoms. **(A)** PPN efferents to brain areas involved in PD motor symptoms, **(B)** PPN efferents contacting brain areas involved in PD-related sleep disturbances. STR, Striatum; THAL, Thalamus; STN, Sub-thalamic nucleus; GPi, Globus Pallidus Interna; DRN, Dorsal Raphe Nucleus; Med, Medulla; SNr, Substantia Nigra Pars Reticulata; SNc, Substantia Nigra Pars Compacta; H-Thal, Hypothalamus; (Images modified from serviermedical art https://smart.servier.com).

The pedunculopontine tegmental nucleus (PPN), a heterogeneous brainstem structure, consists of glutamatergic, cholinergic, GABAergic, and glycinergic neurons (Mineff et al., 1998; Wang and Morales, 2009; Pienaar and van de Berg, 2013), which co-express neuropeptides including nitric oxide, substance P, atriopeptin, NADPH diaphorase, calcium binding proteins, and corticotropin-releasing factor (Vincent et al., 1983; Standaert et al., 1986; Austin et al., 1995; Martinez-Gonzalez et al., 2012; D’Onofrio et al., 2015). Co-expression of acetylcholine (ACh) with GABA or glutamate has also been reported (Lavoie and Parent, 1994; Wang and Morales, 2009). The PPN is functionally divided into two components, a rostral portion containing GABAergic, glutamatergic, and sparse cholinergic neurons sending projections primarily to motor structures including the substantia nigra and thalamus, and a caudal portion consisting mainly of cholinergic and glutamatergic neurons projecting to structures involved in reward such as the nucleus accumbens (Dautan et al., 2016; Xiao et al., 2016). PPN neurons also send descending projections to cerebellar nuclei, brainstem structures like the pontinus oralis, gigantocellular nucleus, and the spinal cord to modulate movement and muscle tone (Rye et al., 1988; Spann and Grofova, 1989; Martinez-Gonzalez et al., 2014). See **Figures 1A, B** for PPN efferent projections and their involvement in Parkinson’s disease (PD) symptoms.

### PPN in PD

PD is associated with degeneration of nigrostriatal dopamine (DA) neurons; however, PPN cholinergic, glutamatergic, substance P, GABAergic, and glycinergic neurons also degenerate in PD (Hirsch et al., 1987; Jellinger, 1988; Gomez-Gallego et al., 2006; Rinne et al., 2008; Pienaar and van de Berg, 2013). Microglial activation and inflammatory responses occurring in PD likely contribute to PPN neurodegeneration (Elson et al., 2016). Cholinergic loss, in particular, is linked to PD symptom severity, especially akinesia and gait deficits (Pahapill and Lozano, 2000; Rinne et al., 2008; Bohnen et al., 2013). Multiple factors likely contribute to death of cholinergic PPN neurons in PD. For instance, mitochondrial DNA deletion occurs more often in PPN cholinergic neurons than in other PPN neurons (Bury et al., 2017). If substantial, this may inhibit protein translation and energy production. In addition, alpha synuclein aggregates may differentially affect PPN neuronal subtypes, as glutamatergic and cholinergic PPN neurons show more effects of alpha synuclein aggregates than GABAergic neurons (Heinrich et al., as cited by Surmeier, 2018).

Furthermore, it is unknown whether cholinergic loss is a downstream response to prior death of monoaminergic neurons, or whether death of PPN cholinergic neurons precipitates loss of DA neurons (Bensaid et al., 2016; MacLaren et al., 2018). Past research in preclinical models indicates that both processes may occur. Additionally, lesioning both nigrostriatal dopamine and PPN cholinergic systems simultaneously results in more severe degeneration (Bensaid et al., 2016). These results imply that the reciprocal connections shared by PPN cholinergic and nigrostriatal DA neurons may be critical to their survival, and that simultaneous lesion alters cellular activity, potentially contributing to neurodegeneration. Accordingly, following nigrostriatal DA lesion, compensatory increases in activity occur in PPN neurons (Palombo et al., 1990; Carlson et al., 1999; Breit et al., 2001; Zhang et al., 2008). Initial or sustained hyperactivity of PPN glutamatergic or subthalamic nucleus glutamatergic neurons may accelerate nigrostriatal and PPN neurodegeneration and may be exacerbated by simultaneous loss of PPN cholinergic neurons. PPN hyperactivity may also arise due to loss of nigral DA and raphe serotonin afferents in PD, as PPN neurons contain the inhibitory DA D2R and 5HT1AR (Leonard and Llinás, 1994). Conversely, PPN hypoactivity (Jang et al., 2012) caused by increased SNr and GP inputs may lead to more severe PD symptoms (Nandi et al., 2002). Perhaps, PD heterogeneity could be partially explained by the order of cellular degeneration and by differential PPN activity. Future research should investigate the timing of neuronal loss, affected projections, and behaviors resulting from cell loss in these two lesion paradigms.

Although under-investigated, a recent study in preclinical models indicates that galanin may rescue PPN cholinergic neurons in PD. Following SNc lactacystin lesion, galanin is upregulated in rostral PPN cholinergic neurons (Elson et al., 2018). Galanin inhibits cholinergic transmission and may be neuroprotective, as it guards basal forebrain cholinergic neurons from amyloid beta toxicity in Alzheimer’s disease (Ding et al., 2006; Pirondi et al., 2010; Elliot-Hunt et al., 2011). In PD, galanin could reduce the likelihood of apoptosis in response to Lewy bodies or neuroinflammation (however, see also, Crawley et al., 2002).

### PPN Involvement in Movement

The role of the PPN in generating locomotion remains controversial; however, early studies suggest PPN involvement in gait, an assertion that has been partially supported and further refined based on recent studies. Early research shows that the PPN and cuneiform nucleus make up the mesencephalic locomotor region (MLR), a functional neurocircuit modulating movement, rhythm, and speed upon electrical stimulation (Garcia-Rill et al., 1987; Garcia-Rill and Skinner, 1988; see **Figure 1A** for PPN projections related to PD motor symptoms). MLR projections to the lateral paragigantocellular nucleus (LPGi) modulate activity of motorneurons and ventral spinal laminae “central pattern generators” that regulate patterns of locomotor activity (Grillner and Wallen, 1985). In support of the PPN’s role in gait, fMRI studies demonstrate that PPN activity increases during fast imagined walking in healthy individuals (Bakker et al., 2008; Karachi et al., 2010); however, whether the PPN exerts its effects through ascending or descending pathways in actual fast walking is unknown. Although both PPN glutamatergic and cholinergic neurons may exert effects through LGPi efferents, recent data from preclinical models show that these neurons play different roles in locomotion. Based on these studies, glutamatergic neurons control flexor muscles to stop locomotion and are involved in slow-paced movement and exploratory behavior (Caggiano et al., 2018; Josset et al., 2018), whereas cholinergic neurons control extensor muscles and prolong the stance phase of locomotion and can also increase speed (Roseberry et al., 2016).

Ascending PPN efferents are also well-positioned to influence movement, although most existing data on this subject stem from research in preclinical models. Most importantly, the PPN projects to thalamic nuclei, which guide movements *via* thalamospinal projections. Additionally, although less direct, PPN glutamatergic and cholinergic inputs to SNc and striatum may affect both direct and indirect pathways. Relatedly, PPN cholinergic terminals in SNc drive locomotion (Xiao et al., 2016), whereas the motor outcomes of PPN afferents to striatum remain enigmatic, but are likely modulatory in nature (Dautan et al., 2016; Assous and Tepper, 2019). Finally, PPN cholinergic and GABAergic inputs to SNr exert influence over the nigrostriatal pathway. There, PPN ACh inhibits MSN terminals *via* M4R, inhibiting movement (Moehle et al., 2017), and PPN GABAergic projections improve motor learning through thalamic disinhibition (Li and Spitzer, 2019). Though PPN neurons likely contribute to normal gait, the role of neurons in the basal forebrain and cuneiform nucleus should also be carefully considered (Xiang et al., 2013; Sarter et al., 2014).

### PPN Involvement in PD Gait and Motor Deficits

Nearly 20%–60% of PD patients experience gait dysfunction including freezing of gait (FOG) that is not consistently improved by DA replacement therapy (Giladi et al., 2001). FOG and falls are more common in PD patients exhibiting decreased ACh metabolism and reuptake in the thalamus (Bohnen et al., 2009; Bohnen and Albin, 2011; Bohnen et al., 2019), where the PPN is the main source of ACh. Cell death and synaptic inhibition from the GPi and SNr likely contribute to this decrease in cholinergic tone. In animal models, chemogenetic activation of PPN ACh neurons rescues motor deficits, indicating that cholinergic neurons remain which may be suitable therapeutic targets in PD (Pienaar et al., 2015). Similarly, cholinesterase inhibitors decrease falls in some PD patients (Chung et al., 2010).

Given its role in locomotion, the PPN has been considered as a site for deep brain stimulation (DBS) to improve otherwise intractable postural instability, FOG, and falling in PD and atypical Parkinsonism. Results of this approach have been varied. Multiple studies demonstrate that PPN-DBS can ameliorate FOG or general gait parameters in PD patients (Pereira et al., 2008; Ferraye et al., 2010; Khan et al., 2011; Thevathasan et al., 2011; Khan et al., 2012a; Khan et al., 2012b; Mazzone et al., 2014; Welter et al., 2015; Mestre et al., 2016). Although unilateral (Moro et al., 2010) and bilateral (Ferraye et al., 2010) approaches decrease falls, double-blind studies indicate the superiority of bilateral PPN stimulation for improving PD-related gait symptoms (Thevathasan et al., 2010; Thevathasan et al., 2012). Conversely, postural instability is not consistently improved with PPN-DBS (Moro et al., 2010; Thevathasan et al., 2010). Despite symptomatic improvements in some studies, open questions remain on the clinical relevance of PPN-DBS, as it cannot eliminate PD gait symptoms, and exact mechanisms underlying PPN-DBS effects are unknown. Furthermore, side effects ranging from oscillopsia, paresthesia, and even worsening of FOG have occurred in some patients (Hamani et al., 2007).

Multiple factors explain inconsistent PPN-DBS clinical outcomes. First, electrode placements in the PPN of PD patients vary. MRI placements correlated with patient outcomes suggest bilateral lead placement in caudal PPN is most efficacious for gait improvement (Goetz et al., 2018; Khan et al., 2012b). Relatedly, preclinical data show that rostral PPN lead placement has deleterious effects on movement and that caudal PPN controls stepping behavior (Gut and Winn, 2015; Garcia-Rill, 1986; Garcia-Rill and Skinner, 1988; Garcia-Rill, 1991). Second, stimulation parameters for PPN-DBS differ from one study to another. Many paradigms employ frequencies within beta and gamma ranges, but some have also employed stimulation at very low frequencies. Finally, small sample sizes, differences in disease progression, and the heterogeneity of PD signs and symptoms likely contribute to differential results regarding PPN-DBS.

### PPN Contribution to L-DOPA Response

DA replacement therapy with L-DOPA is standard treatment for PD. However, chronic use results in debilitating abnormal involuntary movements termed L-DOPA-induced dyskinesia (LID) in up to 90% of PD patients (Ahlskog and Muenter, 2001). Severe LID can lead to hospitalization and reduced quality of life (Péchevis et al., 2005; Lyoo and Lee, 2011). Although the PPN is not well investigated for its role in L-DOPA’s motor efficacy and in LID, existing evidence from patients undergoing PPN-DBS and preclinical evidence on the PPN’s involvement in motor stereotypy suggest that the PPN may contribute.

For instance, PD patients decrease their L-DOPA dose following long-term treatment with PPN-DBS (Mazzone et al., 2014), suggesting that PPN-DBS has pro-motor effects and may potentiate L-DOPA’s motor efficacy. Prior combination of PPN-DBS with L-DOPA shows additional motor benefit beyond L-DOPA alone (Jenkinson et al., 2008; Stefani et al., 2007). Additionally, combining caudal PPN-DBS with L-DOPA in preclinical models results in attenuation of LID (Gut and Winn, 2015). Similarly, immediate early genes associated with development and expression of LID are upregulated in the PPN of dyskinetic rodents (Bastide et al., 2014), implicating the PPN in LID expression.

The PPN has long been known to contribute to motor stereotypy in preclinical models; contextualizing this literature may provide useful insights into the role of the PPN in LID, as research on the PPN’s contribution to LID is sparse. PPN lesion produces orolingual stereotypy in response to d-amphetamine and apomorphine (Inglis et al., 1994), implying that PPN neurons suppress stereotyped movements in the normal brain. Furthermore, existing evidence shows that PPN GABAergic and cholinergic neurons have differing effects on motor stereotypy. PPN infusion of GABAergic antagonists reduces motor stereotypy produced by apomorphine and SNr microinfusion of muscimol (Childs and Gale, 1983), intimating that PPN GABA projections inhibit SNr neurons to promote motor stereotypy, or that SNr GABA projections to PPN promote motor stereotypy. Conversely, PPN infusion of mAChR antagonists increases motor stereotypy and exacerbates LID (Mathur et al., 1997; Chambers et al., 2019). Taken together, these results suggest that decreasing GABAergic tone or increasing cholinergic tone in the PPN may decrease LID, but this must be examined further. If true, this provides further support for hypoactivity of the PPN in PD. Future research should address specific contributions of PPN neuronal subtypes, and investigate the role of PPN efferents to the SNr and striatum.

### PPN Involvement in Sleep

The PPN is part of the reticular activating system (RAS), a group of nuclei regulating consciousness and attention. The PPN receives lateral hypothalamus orexin projections and SNr GABA projections that regulate muscle tone during sleep and wakefulness (Takakusaki et al., 2011, see **Figure 1B**). As mentioned previously, SNr and GPi GABA transmission to PPN is enhanced in PD. Importantly, when GABA B receptors are bound, REM sleep is inhibited (Sakai and Koyama, 1996; Ulloor et al., 2004; Datta, 2007). This alone may account for a significant portion of REM sleep problems in PD.

PPN cholinergic neurons promote EEG desynchronization and changes in state of consciousness *via* thalamic projections which regulate cortical activity (Hallanger et al., 1987; Steriade et al., 1990; Kobayashi et al., 2002; Mena-Segovia et al., 2008; Kotagal et al., 2012). PPN cholinergic neurons are active during REM sleep and wakefulness, manifesting alpha activity at rest and gamma oscillations related to behavior during waking (Steriade et al., 1991; Steriade et al., 1996; Kezunovic et al., 2011). During REM sleep, cholinergic neurons promote muscle atonia through projections to the subcoeruleus dorsalis (Baghdoyan et al., 1984; Sanford et al., 1994), and nucleus reticularis gigantocellularis neurons which project to motorneurons (Takakusaki et al., 2011). Cholinergic neurons promote REM and pontogeniculooccipital waves characteristic of REM sleep *via* dorsolateral geniculate nucleus and frontal eye field efferents (Sakai et al., 1976; Callaway et al., 1987; Shouse and Siegel, 1992; Rye, 1997). During slow wave sleep cholinergic neurons also promote nested gamma oscillations associated with memory replay and neuronal plasticity in cortex and hippocampus (Lee and Wilson, 2002).

### PPN Involvement in PD-Related Sleep Dysfunction

Sleep disturbances resulting in excessive daytime sleepiness occur in 98% of PD patients (for review see Comella, 2007). While prevalence of PD-related sleep disorders alone is concerning, sleep dysfunction, particularly REM sleep behavior disorder (RBD), is linked to increased risk of cognitive impairment in PD patients (Marion et al., 2008; Gagnon et al., 2009). RBD is present in an estimated 50% of PD patients and is characterized by lack of muscle atonia during REM sleep which leads to patients engaging in complex motor behaviors during sleep. Intriguingly, diagnosis with idiopathic RBD often precedes PD and other synucleinopathies and may represent a prodromal phase of these illnesses (Boeve et al., 2001).

PPN ACh and substance-P-expressing neurons may contribute to the pathogenesis of RBD. Despite similar age of onset and disease duration, PD patients with RBD show decreased cholinergic transmission in cortex and thalamus relative to PD patients without RBD (Kotagal et al., 2012; however see also Bedard et al., 2019). Similarly, AChE inhibitors decrease symptoms of RBD, meaning that RBD may arise due to loss of cholinergic tone (Ringman and Simmons, 2000). Substance P is co-expressed in 27% of caudal PPN ACh neurons and is co-released onto the pontoreticular formation, where it has additive effects beyond those of ACh on initiation and maintenance of sleep (Kohlmeier et al., 2002). Substance P neurons degenerate in PD, which likely contributes to REM sleep atonia in PD patients. Congruent with PPN involvement in sleep dysfunction, PPN-DBS increases sleep efficiency, REM and stage 2 sleep, and decreases awakenings in PD patients (Romigi et al., 2008). Although understudied, these findings demonstrate that PPN-DBS could be used in the future to improve sleep for PD patients.

### Therapeutic Strategies/Future Directions

Interpreting PPN-DBS outcomes is impeded by small sample sizes, differences in electrode placement, stimulation paradigms, and disease progression. Additionally, PPN-DBS paradigms employ open-loop DBS which does not consider the situation and/or symptoms the patient is experiencing, nor brain activity in other areas which influence PD symptoms. Employing closed-loop PPN-DBS may be more effective (Rosin et al., 2011; Hebb et al., 2014; Sun and Morrell, 2014), as different stimulation frequencies have varying effects on PD symptoms. Using machine learning strategies, clinicians could correlate specific patterns of EEG readout with problems with FOG or other symptoms, and then use this information to promote more efficacious PPN-DBS. Future research should also address effects of exercise on the PPN, as recent data show that similar to hippocampal and basal forebrain cholinergic neurons (Molteni et al., 2008; Hall and Savage, 2016), exercise may induce PPN neuroplasticity beneficial for motor learning (Li and Spitzer, 2018). Finally, gene-therapy strategies focusing on neuroprotection through galanin or modulating L-type calcium channels should be considered, as well as gene therapy strategies that target specific PPN projections for symptomatic relief (see **Table 1**).

**Table 1:** Potential targets for PPN-mediated improvement of PD motor and non-motor symptoms.

PPN Afferent/Efferent Projection	Symptom(s)	References
**Intralaminar and Ventromedial Thalamus (Efferent)**	Motor Deficits (Freezing of gait, falling) REM Sleep Behavior Disorder	(Bohnen et al., 2009; Hallanger et al., 1987; Bohnen and Albin, 2011; Brazhnik et al., 2016; Bohnen et al., 2019; Kezunovic et al., 2011; Steriade et al., 1990; Mena-Segovia et al., 2008)
**Globus Pallidus Interna (Afferent)**	Akinesia, freezing of gait	(Nandi et al., 2002; Zhang et al., 2012)
**Lateral Paragigantocellular Nucleus (Efferent)**	Trouble initiating movement	(Garcia-Rill et al., 1987; Garcia-Rill and Skinner, 1988; Roseberry et al., 2016; Josset et al., 2018)
**Lateral Reticular Thalamic Nucleus, Superior Colliculus, Frontal Eye Fields (Efferent)**	Visual Sensorimotor Integration/Oscillopsia resulting from PPN-DBS	(Redgrave et al., 1987; Wallace and Fredens, 1988; Okada and Kobayashi, 2009)
**Striatum (Efferent)**	Motor Symptoms (akinesia) L-DOPA-induced dyskinesia	(Dautan et al., 2016; Assous and Tepper, 2019)
**Substantia Nigra Pars Reticulata (Efferent)**	L-DOPA-induced Dyskinesia REM Sleep Behavior Disorder	(Childs and Gale, 1983; Moehle et al., 2017)
**Vestibular System (Afferent)**	Postural Instability	(Cai et al., 2018; Vitale et al., 2011)

## Conclusion

Overall, through innervation of ascending and descending motor structures, RAS, and cortex, the PPN is well-positioned to modify PD motor and sleep symptoms. However, to develop effective PPN-centered treatment strategies, there is a need for cell-specific manipulations in the PPN. Currently, this is challenging, as the contribution of specific PPN pathways to gait and RBD symptoms, and pathways that govern PPN’s modulation of L-DOPA’s effects are not well understood. Additionally, receptor types specific to each PPN neuron and how they change in health and disease are not well-defined. Therefore, future research should elucidate the contribution of specific PPN afferent and efferent connections to behavior and identify receptors exclusive to each type of PPN neuron. Additionally, as PD is a heterogeneous disorder, it is especially important that the therapeutic strategies employed are custom-tailored to the patient based on his or her symptoms.

## Author Contributions

Contributed to writing of the manuscript: NC, CB Created figures: KL.

## Funding

American Parkinson’s Disease Association Research.

## Conflict of Interest

The authors declare that the research was conducted in the absence of any commercial or financial relationships that could be construed as a potential conflict of interest.
